# A Systematic Review of Dementia Research Priorities

**DOI:** 10.1177/08919887241232647

**Published:** 2024-02-09

**Authors:** Manonita Ghosh, Pelden Chejor, Melanie Baker, Davina Porock

**Affiliations:** 1 Social Ageing (SAGE) Futures Lab, School of Arts and Humanities, 2498 Edith Cowan University, Perth, WA, Australia; 2 Centre for Research in Aged Care, School of Nursing and Midwifery, 2498 Edith Cowan University, Perth, WA, Australia

**Keywords:** alzheimer’s disease, dementia, geriatrics, behavioural disturbances, cognitive decline, health services research

## Abstract

**Introduction:**

Patient involvement is a critical component of dementia research priority-setting exercises to ensure that research benefits are relevant and acceptable to those who need the most. This systematic review synthesises research priorities and preferences identified by people living with dementia and their caregivers.

**Methods:**

Guided by Joanna Briggs Institute methodology, and Preferred Reporting Items for Systematic Reviews and Meta-Analyses framework, we conducted a systematic search in five electronic databases: CINAHL, Medline, PsycINFO, Web of Science and Scopus. The reference lists of the included studies were also manually searched. We combined quantitative and qualitative data for synthesis and descriptive thematic analysis.

**Results:**

Eleven studies were included in this review. Findings are grouped into four main categories: Increase in knowledge, education, and awareness; Determining the cause; Sustainability of care; and Cure of dementia and related conditions.

**Conclusion:**

There is a need to respond to the stigma associated with dementia, which limits access to care and the quality of life for both people living with dementia and their caregivers. We need to work on changing public, private and workplace attitudes about dementia and encourage supporting and participating in dementia research. Future research should involve people living with dementia and their primary caregivers from culturally and linguistically diverse communities in priority-setting exercises.

## Introduction

It has been six years since the World Health Organisation (WHO) developed the Global Action Plan 2017-2025 to address dementia challenges for those affected, namely people living with dementia, families, and health care systems.^1^ However, with less than two years left to reach the goal, progress is still far from meeting the target. Countries across the world find it challenging to take practical steps to ensure that their policy plans align with the Global Action Plan to address dementia^2^ and so Alzheimer’s Disease International has urged WHO to extend the Global Action Plan’s deadlines by four years, until 2029.^3^ With over 55 million people living with dementia worldwide, and nearly 10 million new cases annually, it stands as the seventh leading cause of death, highlighting its significance as a recognised public health concern.^4^ Yet dementia research continues to be underfunded, receiving only one-eighth of the funding allocated to cancer research.^5^ Providing evidence-based care is a challenge for many countries due to the scarcity of research funding. Research priority-setting exercises are helpful in developing a dementia research plan and has been defined as “a collective activity for deciding which uncertainties are most worth trying to resolve through research; uncertainties considered may be problems to be understood or solutions to be developed or tested; across broad or narrow areas.”^6^

Patient and public involvement is a critical component of research, including research priority setting. Involvement means that research is ‘carried out ‘with’ or ‘by’ members of the public rather than ‘to,’ ‘about’ or ‘for’ them.’^7^ This is to ensure that research benefits, and is relevant and acceptable to, the population it seeks to address; in addition to improving the quality of research overall.^8^ The inclusion of people living with dementia in aged care research is a potentially neglected area due to the difficulties of engaging with people experiencing a cognitive decline.^9^ However, involving people with dementia in a research and research priority exercise is challenging, and often restricted by rules and regulations in many countries including Australia. To provide pathways for the participation of older adults who do not have the capacity to consent in health and medical research, in 2020 the Western Australian Department of Health amended the Guardianship and Administration Act 1990.^10^ The amendment mandated that an independent medical practitioner determines the suitability of each potential research participant and that the lead researcher is a medical practitioner.^10^ The outcome of the amendment makes it more difficult to involve people with dementia due to the logistics of meeting these requirements including the cost of an independent medical review in a contemporary, resource-limited research environment. It also means that, being led by a medical practitioner, is conducted through a medical lens minimising the impact of other health and social research frameworks. This systematic review examines studies that involved people living with dementia and their caregivers to synthesise research priorities and preferences identified by people living with dementia and their caregivers.

## Methods

The Joanna Briggs Institute (JBI) methodology for systematic reviews,^11^ and the Preferred Reporting Items for Systematic Reviews and Meta-Analysis^12^ (Supplemental document 1) guided this review to systematically identify the research priorities and preferences of people with dementia and family caregivers. We followed the eight-stage JBI framework for systematic review: (1) formulating a review question, (2) defining inclusion and exclusion criteria, (3) locating studies through searching, (4) selecting studies for inclusion, (5) assessing the quality of studies, (6) extracting data, (7) analysing and synthesising the relevant studies, and (8) presenting and interpreting the results, potentially including a process to establish certainty in the body of evidence. Systematic reviews do not involve human participants and therefore ethical approval is not required. The study protocol was registered in PROSPERO (CRD42022346466).

### Search Strategy

**Figure 1.**
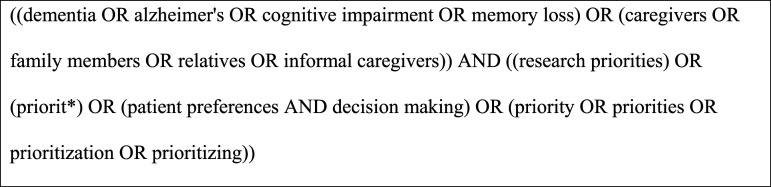
Medline search strategy.

A systematic search using an a priori search strategy was conducted in CINAHL, Medline, PsycINFO, Web of Science, and Scopus on 7 July 2022. We developed the search strategy with support from a subject librarian and used a three-step search strategy: first, we identified keywords by an initial limited search of two databases CINAHL and Medline; second, we refined the search terms; third, we searched all included databases and Google scholar; and fourth, manually searched the reference list of identified reports and articles. A combination of the following keywords in either title or abstract or MeSH term was used based on key concepts of dementia and research priority: dementia OR Alzheimer’s disease OR cognitive impairment OR caregivers OR carers OR “formal carers” OR “informal carers” AND “research priorit*” OR priority-setting OR “priorities for dementia care” OR “patient preference” OR preferences (Figure 1).

### Eligibility Criteria

We used the Population, phenomena of Interest, and Context (PICo) framework to determine which articles were eligible for inclusion according to JBI guidelines.^13^ The eligibility criteria comprised research studies involving people with dementia and family caregivers, with or without the involvement of other relevant stakeholders (Population); and studies conducted to identify dementia research priorities and preferences (Interest). Studies conducted in any setting including home care, residential care, community care, and respite care were eligible (Context). Studies employing either qualitative or quantitative research methods were included. Only those studies published in English met our inclusion criteria for feasibility purposes. Excluded were studies that did not incorporate individuals living with dementia and caregivers in the process of priority setting. Literature reviews, conference abstracts, commentaries, editorials, letters, news, and opinion papers were also excluded.

### Study Selection

All identified studies were imported into EndNote X9.3.3 (Clarivate Analytics, Philadelphia, USA) and duplicates were removed. PC and MG screened references by titles and abstracts using the Rayyan software,^14^ and potentially relevant articles were retrieved for full-text assessment. Three reviewers (PC, MG, MB) independently assessed the eligible full-text articles in detail against the inclusion criteria, recording the reasons for the exclusion of studies. Disagreements between the reviewers at each stage of the selection process were resolved through discussion.

### Quality Appraisal

Quality appraisal for the qualitative and quantitative included studies was conducted using JBI Critical Appraisal Checklists for mixed methods studies.^11,13^ Three reviewers (PC, MG, MB) assessed all the articles included for review for methodological quality. Disagreements between the reviewers were resolved through discussion.

### Data Extraction and Synthesis

Data relating to the characteristics and the findings of included studies were extracted by three independent reviewers using the JBI data extraction tool. The data extracted included the author, year, and title of the article, the aim of the study, study participants, primary data collection method and data analysis, and top research priorities and preferences. Our data analysis focused on “what are the research priorities and preferences identified by people living with dementia and their caregivers which aligned with a question in PROSPERO. Following the Braun and Clarke approach,^15^ we used descriptive and narrative thematic analysis to analyse the data. We also utilised a convergent integrative approach,^16^ which combined quantitative and qualitative data for the analysis. In the convergent integrative approach, qualitative data were extracted verbatim and quantitative data were converted into qualitative form ascribed as qualitised. The qualitative and qualitised data with similar meanings were then grouped into several narrow categories, synthesised, and analysed for key themes. Where textual pooling was not possible, we explained them in a narrative way.

## Results

### Literature Search

**Figure 2.**
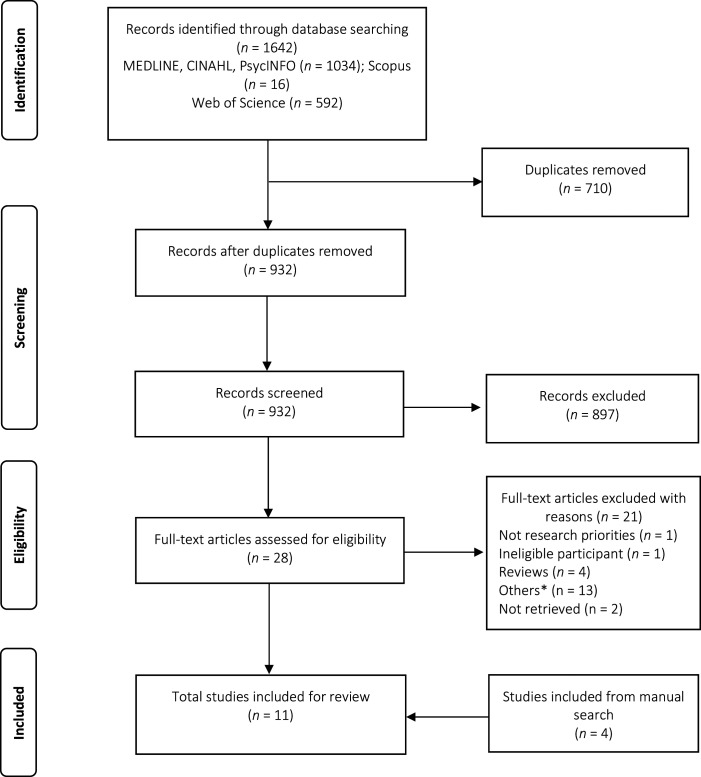
Literature search and selection process. *Commentaries, editorials, letters, conference proceedings.

A total of 1642 studies were identified from the database of which 710 duplicate articles were removed. Of the 932 studies screened for titles and abstracts, only 28 studies were included for full-text review. A total of 11 studies were included in this review (7 of them after the full-text assessment and 4 from Google Scholar and a manual search of the reference list of the included article). The overall literature search and study selection process is outlined in Figure 2.

### Study Characteristics

Of the 11 studies included in this review, five were conducted in the United States of America, three were in the United Kingdom, two in Canada and one in Australia. Most studies undertook an extensive community and/or stakeholder consultation process through Delphi or James Lind Alliance research methods and comprised of the development of advisory groups, interviews, focus groups, and surveys and established consensus for the research priorities among stakeholders. Yet, Delphi and James Lind Alliance’s methods are primarily quantitative in approach. Two studies, Armstrong et al^17^ and Frank et al^18^ employed qualitative interviews and focus group discussions and provided thematic and narrative analysis of the data often with direct quotes from research participants. There was another study by Law et al^19^ which used a quantitative survey questionnaire to gain 514 responses and reported findings by percentage. Despite the differences in the research methods used for data analysis, research priorities and preferences by the people with dementia and their caregivers were found to be common across the studies. Among the studies, four^17,18,22,26^ specifically focused on individuals living with dementia and caregivers, while the remaining studies also included researchers, clinicians, and other service providers.

Although all studies involved consumers and stakeholders including people living with dementia, family caregivers, community members and/or researchers, clinicians, and other health professionals, some did not mention the number in each group and/or the age of the participants. In those papers that reported the sample size, there were substantial differences ranging from 12 to more than 1500 participants. An overview of the studies including methods, number of participants with dementia and caregivers, is presented in Table 1. Research priorities and categories overlapped across studies. Only two studies focused on identifying gaps and research priorities in culturally and linguistically diverse communities.^20,21^

**Table 1.** Study Characteristics for the Studies Identifying Priorities for Dementia Care Research.

Author, Year & Country	Aim of Study	Participants	Data Collection Methods and Analysis	Findings
Armstrong et al, 2020, USA	To identify research priorities of people living with lewy body dementia and caregivers.	20 people living with lewy body dementia and 25 family caregivers.	Semi-structured interviews over the telephone. A qualitative descriptive approach was used to analyse transcripts and identify themes and subthemes.	Nine research priorities with several subthemes for each topic were identified.
Bethell et al, 2018, Canada	To identify and prioritize questions for research related to living with dementia, prevention, diagnosis, and treatment.	Survey (1217 individuals and groups). Interim prioritisation workshop (249). Final prioritisation workshop (28 people living with dementia, friends/family caregivers, health care and service providers).	James lind alliance method was used. 79 research questions were distributed and ranked online and via surveys. 23 top priorities were compared with other international initiatives.	Top 10 priorities were identified.
Frank et al, 2020, USA	To report the formation and implementation of the stakeholder group, and it’s influence on the research contents.	12 people living with dementia including frontotemporal degeneration, lewy body dementia, alzheimer, and mixed dementia.	Group discussions. Research recommendations and information were thematically analysed.	Identified 58 research recommendations including 30 ideas from people living with dementia group.
Kelly et al, 2015. UK	To identify and prioritise top 10 unanswered questions around prevention, treatment, diagnosis, and care of dementia.	Survey (64 people living with dementia, 1188 family carers, 16 caregivers, along with other healthcare professionals). Face-to-face workshops (2 people living with dementia and 5 caregivers).	James lind alliance method was used. Survey responses were thematically analysed. Research questions were developed, refined, and formatted followed by face-to-face prioritisation workshops.	Identified top 10 research priorities.
Law et al, 2011, UK	To identify dementia research priorities, location, type of research and desired outcome measures of people living with dementia and caregivers.	514 people with dementia, their caregivers, and researchers completed the survey. The number of people with dementia and their caregivers not reported.	A survey with 15 topics distributed and topics prioritised. Quantitative analysis was used to compare difference in the priority topics between the 3 groups.	Identified top four important topics, which were identical across all 3 groups of respondents.
National ageing research institute, 2020, Australia	To identify research and research translation priorities and gaps in dementia for cultural and linguistically diverse culturally and linguistically diverse communities.	The number of people with dementia and their caregivers not reported. Researchers, service providers, aged care workers, community members, older people, people with dementia and caregivers were involved, in addition to community consultations with people over 65 from migrant communities.	Several consultations, two stakeholder workshops, 19 nationals culturally and linguistically diverse community consultations, a national online survey, and a modified delphi process. Themes were identified and consensus was established.	Identified 21 priority research and translation need.
Patel et al, 2021, USA	To identify research priorities, and gaps in care for people living with dementia and family caregivers.	23 people living with dementia, 101 family caregivers, and 62 health and social care professionals.	Consultations, stakeholder advisory council inputs, symposium, webinars, and online learning community. Community members and professionals prioritised research topic distributed via online survey. People living with dementia completed the survey face-to-face via zoom.	Identified 46 priority dementia care topics. Support for continuing to live in their own homes was ranked the highest priority.
Porock et al, 2015, USA	To identify areas of consensus on priorities for public policy and research priorities funded by public policy	First survey 388, and second survey 301 people living with dementia or family caregivers.	Delphi surveys. Content analysis was used to group responses from round-1 delphi survey into thematic areas for potential priorities. In round-2, participants ranked the identified topics in terms of importance. Calculation of consensus ranking was performed.	Identified 11 topic areas - 5 priorities for government, and 6 priorities for research.
Shah et al, 2016, USA	To identify research priorities to reduce the global burden of dementia in the next 10 years.	201 participants - researchers, people living with dementia and family members from 39 countries. Another 162 individuals including 18 caregivers of people living with dementia and 1 people living with dementia participated in prioritisation workshop.	Delphi method was used. Respondents were asked to send 3 to 5 research questions. Content analysis was used to group responses into thematic areas for potential priorities. In round 2, identified topics were ranked in terms of importance. Statistical analysis was conducted for scoring.	Top 10 research priorities identified.
Stolee et al, 2011, Canada	To describe the process implemented to identify research priorities for Alzheimer’s disease and related dementia.	Focus group (11 researchers, 16 clinicians, 4 people with early-stage dementia and 23 family caregivers). Surveys (60 researchers, 106 clinicians, 23 family caregivers and people living with dementia). Additional 53 researchers, clinicians and representatives of policy and planning participated in workshops.	Methods included environmental scan, interviews, focus groups, surveys, and consensus workshop. Interview and focus group data were thematically analysed to develop the survey research items with likert scales to examine and confirm the themes with a larger sample.	Consensus statement on research priorities were grouped under 3 themes.
Tierney et al, 2021, UK	To identify research ideas and priorities for a migrant community.	Eight people living with dementia and their 17 family members with musculoskeletal and mental health condition from South Asian migrant communities.	Data collected through multisite collaboration and discussion, and consensus gained in workshops where employed artists produced pieces of art based on priorities for research identified by each group. Group discussions were thematically analysed.	Research priorities grouped into three categories: Young onset dementia, women and people with musculoskeletal and mental health condition.

### Research Priorities

We categorised the research priorities into 4 main themes and corresponding subthemes as outlined in Table 2. These themes and subthemes demonstrate interconnectedness and interdependence.

**Table 2.** Research Priority Areas.

Research Priorities
Increase in knowledge, education, and awareness
Symptoms, cause, risk factors, diagnosis, prognosis, nutrition, and treatment.
Impact on health and well-being, family, and relationship.
Information on available local resource and support service.
Stigma.
Determining cause of dementia
Understanding and identification of physiological and molecular mechanisms of dementia.
Understanding and identification of risk factors including genetic and environmental.
Sustainability of care
Early detection, diagnosis, and prevention: identification of early symptoms and development of screening tool, prevention.
Improving impact and outcomes for persons with dementia and the caregivers: quality of life, activity of daily living and independent living, behavioural, mental, and emotional health, employment challenges and end of life.
Supporting family caregivers: burden, quality of life, health, and wellbeing, identifying options to pay for caregiving, employment challenges, balancing caregiving, and employment.
Professional skill development: appropriate training, communication, caregivers training.
Care setting and environment: homecare, hospital, long-term care facilities, day-care.
Development of policy and practice: culturally appropriate person-centred care, consolidating the disease names, implications of financial burden.
Cure for dementia and related conditions
Cure for dementia or dementia related conditions.
Effective pharmaceutical treatment and intervention.
Effective non-pharmaceutical psychosocial therapy and technological intervention.

#### Increase in Knowledge, Education, and Awareness

Both caregivers and participants with dementia prioritise research efforts focused on how to improve knowledge, education, and awareness of the disease. There are many unknowns about dementia across all areas of research, not only for people living with the condition and communities but also for clinicians and researchers. Participants pointed out a need for education in general for people living with dementia, including family caregivers and the public. The dementia-related issues identified were: knowledge and awareness of symptoms; cause and risk factors; diagnosis, types of dementia and various stages of the condition; its impact on mental and emotional health for the person living with dementia and their caregivers; and information on care available, local resources and support services following a diagnosis of dementia. Additionally, there was some evidence that those people diagnosed with the disease wanted information to improve their understanding by talking to other people in the same situation; to hear directly from them about their experiences, what to expect and their coping strategies.^17^ Participants further indicated that education and information were required about managing anxiety, fear, and other emotions related to a diagnosis of dementia,^25^ diet and nutrition and improving standards of care, in addition to the optimal time to shift from private homecare to a professional home care setting.^24^

There was also an emphasis on the need for increased funding to be made available for research to help better understand how the progression of the disease could be delayed,^28^ and the impact of the disease process on caregivers and relationships was of particular interest to many participants, who voiced that research should focus on how a diagnosis of dementia affects the relationships between a person living with dementia and their family members.^24,25^ Stigma and the impact associated with mental health for those diagnosed and their families were also of particular interest to many participants. One person with a diagnosis of dementia highlighted this by asking “Why do people hide it? How can we help them to come out and talk about it?.”^23,28^ Education to enhance such understanding and to address both stigma and stereotypes, that are often associated with dementia, was identified as a priority. The recommendation was made to identify and consolidate all confusing and stigmatising terminologies under 1, widely recognised and accepted general term, as deemed appropriate. It was felt that this would help reduce stigma and promote more effective communication among people living with dementia, families, and the public.^18^

#### Determining Causes of Dementia

There was a consensus among people living with dementia and their caregivers about the need for more research on the causes of the disease and the expected progression. They expressed concerns about how dementia could be prevented, or the progression of the disease slowed and/or halted, if the root cause of the problem was not identified.^17^ The research focusing on the causes must include the identification of the physiological and molecular mechanisms involved^28^ and the development of biomarkers, diagnostic markers and basic research into the disease mechanisms and prognosis.^17,27^ In addition to desiring to know causes in general, participants wanted further research regarding genetic and environmental risk factors including pre-existing health problems, prior injuries, employment hazards and/or any traumatic event which could potentially contribute to a person developing dementia.^17,28^

#### Sustainability of Care

##### Early Detection, Diagnosis, and Prevention

In some studies, participants mentioned that it could be challenging for them to get a diagnosis and medical attention, even when they noticed signs that ‘something’ was wrong. Early identification of symptoms and the development of an early diagnosis and screening tool were prioritised by consumers and stakeholders.^17,26-28^ Additionally, caregivers articulated that the importance of prevention was better than cure as is commonly addressed in other diseases including the promotion of heart health and mental health.^17,26-28^

##### Improving Impact and Outcomes for People Living With Dementia and Their Caregivers

The idea that research should investigate the specific support needed for people living with dementia was evident in the papers reviewed. Participants emphasised the need to focus on the development and testing of interventions that improve behavioural symptoms, mental and emotional health, activities of daily living, independent living and quality of life,^17,26-28^ including people from culturally and linguistically diverse backgrounds.^21^ Participants in 1 study described the need to support people with dementia to engage in positive health behaviours such as good diet and keeping physically active.^22^ In this study, the participants also emphasised the need to address the employment challenges and potential discrimination in the workplace that people with younger onset dementia can encounter.^22^ The need to support people with dementia and their families who are vulnerable and at higher risk for poor health outcomes^25^ was also identified. Similarly, another study reported on the need for research focusing on care for people with advanced dementia, with or without other illnesses, at the end of life.^24^

##### Supporting Family Caregivers

A review of the studies highlighted that dementia not only has a profound impact on the person diagnosed with dementia but also on their caregivers, and that research must address the separate needs of the caregivers. Both of those groups identified the impact of the caregiving role and acknowledged the need for research addressing different realms of caregiving including caregiver burden, stress, anxiety, depression, well-being, quality of life, and balancing formal employment with caregiving, both in the mainstream population^17,22,26-28^ as well as culturally and linguistically diverse communities.^21^ The need for additional research to identify the most effective ways of supporting family caregivers^24^ and/or alternative care options and resources to pay for caregiving needs, and to provide caregiver support and appropriate direction was identified.^17,28^ There was a strong sense that this was critical because if the caregiver is not supported, then their health would suffer, and they would not be able to provide this critical role because they would have to care for themselves.^17^

##### Professional Skill Development

A common finding was that consumers felt the need for professional skill development through appropriate training for all health and social care providers. They felt that health professionals often did not understand the diseases or have the knowledge to contribute constructively to the healthcare of those affected by the disease. Education and professional development in this field were strongly recommended for all healthcare staff involved.^17^ There was also support for this from the view of the clinicians themselves about the lack of and often conflicting knowledge of dementia among them, indicating a need for appropriate professional development training for healthcare professionals.^28^ It was suggested that the level of dementia-related skills and knowledge held and required by healthcare and social-care providers needs to be identified before providing professional training.^23,25^

The need for effective communication^25^ and cultural competency^18,21^ was also identified as crucial for frontline health and care staff so that they can provide appropriate care and meet the specific needs of people living with dementia and their families from diverse cultural and linguistic backgrounds. Hence, effective communication and cultural understanding of dementia is recommended to be included in the relevant professional training. Participants further indicated the need for effective communication training for caregivers, which would enable them to communicate more effectively and to better take care of both the people living with dementia and the caregivers themselves.^17,25^ There was a suggestion that a ‘caregiver course’ would be helpful and should include how to care for caregivers themselves.^17^

##### Care Setting and Environment

People living with dementia and their family caregivers identified a need for the improvement of the care setting and environment through the creation of dementia-friendly surroundings at both the housing and neighbourhood levels.^23,24^ Indeed, more than 91% of consumer participants ranked as the highest priority research focusing on support, services, and effective interventions for people living with dementia to live in their own homes and to receive quality care.^25^ Care settings and environments also included long-term care facilities, day-care, and hospitals to provide optimal care,^24,26^ however, funding was found to be lacking for home care services,^28^ and there was a need to study the quality of care and the impact of care differences on the people living with dementia and their caregivers, and in multiple settings.^24,25^

##### Development of Policy and Practice

Studies emphasised the need for policy and practice development to ensure implementation, sustainability, and delivery of person-centred care that provides a more coordinated approach,^18,23^ is tailored to people’s culture and language, and is accessible for the individual and the family, regardless of geographic location.^18,21,25^ As touched upon previously in this discussion, the studies suggest the consolidation of disease names for all memory disorders under 1 general term, for example, dementia or Alzheimer’s disease, for ease of advocacy, managing stigma, reporting prevalence and the role of other services.^18,28^ Implications of financial burden on diagnosis, treatment, care support and research were further identified. Hence, recommendations were provided to identify ways to boost funding to increase the number of skilled health and social care providers and to improve workforce issues.^18,23,26^ Studies also identified priorities for dementia research related to capacity building and sustainability to meet the health and social care needs of people living with dementia and their friends or family caregivers and care partners.^23^

#### Cure for Dementia and Related Conditions

Currently, with no known cure for dementia or dementia-related conditions, including Alzheimer’s disease and young onset dementia, the participants emphasised the utmost need for research focusing on the development and trial of treatment and programs that would cure or at least slow cognitive decline.^19,23^ Patel et al^25^ and Armstrong et al^17^ identified that more than 76% of family caregivers prioritised research needs in the development of interventions to slow cognitive decline and to cure the disease.

Participants highlighted the importance of focussing research not only pharmacologic treatment or therapies, but also on drug intervention with minimal side effects and non-pharmacological, psychosocial treatment, and complementary therapies that include supplements, remedies, and alternative treatments.^25,26,28^ Hence, effective approaches other than or in addition to pharmacological treatment were strongly recommended to manage behavioural symptoms of dementia. Indeed, one study elicited 863 research questions from 201 participants and consolidated the questions into 59 thematic research avenues, which were then scored anonymously by 162 researchers and stakeholders from 39 countries, including 11% caregivers and 1% individuals with dementia.^27^ The top thematic research avenues in that study emphasised that high priority should be given to psychosocial intervention research that explores models of care in the community and across the disease progression, including late life and end-of-life care. Similarly, in their study^25^ included 75% of family caregivers who emphasised a non-pharmacological approach to manage behavioural symptoms, since they understood the benefits of cognitive stimulation activities such as games and crosswords in delaying the onset or slowing the progression of dementia.^25^ Participants also emphasised on research focusing on the development and uptake of evidence-based treatments and technological intervention, which are specific for various stages of dementia, dementia subtypes,^28^ and culturally appropriate^21^ in primary and acute care settings.

## Discussion

A careful and rigorous approach to this review of research was taken to identify research priorities from the perspectives of people living with dementia themselves and their family caregivers. We found that research priorities from their perspectives were overlapped and could be grouped into four main categories: Increase in knowledge, education, and awareness; Determining the cause; Sustainability of care; and Cure of dementia and related conditions. Various stakeholder groups including caregivers, clinical and allied health professionals, the public and governance must work collaboratively for the wellbeing of the people living with dementia. We identified only 11 articles which met our inclusion criteria, indicating a limited body of published work on this particular topic. Logan and associates^29^ recently conducted a systematic review similar to ours, although not registered on PROSPERO. They categorised priorities under eight research themes: caregivers, support, awareness and education, drugs and interventions, diagnosis, pathology, research design and prevention – a resonance with our own findings. The papers included in Logan and associates largely overlapped with studies included in our review, however, our approach to data analysis is different to Logan et al review. They used the nine Common Themes of Good Practice (9CTGP) and the Reporting Guideline for Priority Setting of Health Research (REPRISE) checklists to evaluate methodological and reporting quality in their report. They also provided less detail in their analysis of research priorities. In our review, instead of comparing the methods of priority setting exercise, we focused on the analysis of the research priorities by people living with dementia and their caregivers as reported in the included studies aligning with PROSPERO guidelines for this analysis. Further, to align with the objectives of our review, we included studies which employed qualitative study design for priority exercise. For example, Armstrong et al^17^ used semi-structured interviews over the telephone, and a qualitative descriptive approach for thematic data analysis. Similarly, Frank et al^18^ had qualitative focus group discussions as prioritisation methods. In our review, we used a thematic analysis approach of data similar to Logan et al review. However, we reported the findings in qualitative narrative format whereas, Logan reported the research priorities by percentage. We also provided direct quotes from participants reported in the articles. We found that using JBI tools was more appropriate for assessing methodological quality and data analysis in our review.

Similar to what Logan et al^29^ reported, most studies in our review used either Delphi or James Lind Alliance methods, which are primarily quantitative in approach. Only 2 studies^17,18^ took qualitative approaches. Interestingly, the findings were the same irrespective of the research method, but the qualitative study gave more voice to individuals living with dementia and gave them the opportunity to express their views in their own words. The themes of the research priorities and preferences in our review were interrelated and interdependent. In summarising the preferences of people living with dementia and their caregivers, this review captures research priorities and strategies to enhance research efforts that increase knowledge about the cause and cure of dementia, and to improve care and support for individuals with dementia, their families, and caregivers. Our findings of research preferences in knowledge, education, and awareness; early detection, diagnosis; prevention; and sustainability of care were also reported by Logan et al^29^ Many studies in our review emphasised the importance of non-pharmacological treatments and psychosocial support programs as priorities. These findings resonated with a study by Emrich-Mills,^30^ where the top 10 priorities focused on psychosocial support, care, and mental health in dementia. The impact of stigma associated with dementia and education required to address stigma were also found highest rated priorities in Logon et al review.

We synthesised research priorities and preferences identified by people living with dementia and caregivers, which led us to include studies involving individuals with dementia and caregivers with or without the involvement of other relevant stakeholders. Our review acknowledges the difficulties experienced by the studies in involving people living with dementia and caregivers. However, from what was ascertained, there were significant synergies with the views of both people living with dementia and informal caregivers. Based on our findings, involving individuals with dementia and family caregivers contributed to a more comprehensive and informed understanding of the needs of people with dementia and their families. Armstrong et al^17^ noted that despite supporting and prioritising research, individual with dementia and caregivers in their study proposed some topics that fell outside the scope of national research priorities. They reported that individual with dementia and caregivers described research topics aimed at handling symptoms by families, improving daily life, quality of life, and caregiving for person with dementia and their families, whereas national research priorities aimed to inform prevention and effective treatment of dementia. Similarly, in Patel et al’s^25^ study, research emphasising support for continuing to live in their own homes were ranked as the highest priority for people living with dementia. Caregivers, prioritised research aimed at interventions to slow cognitive decline, whereas health professionals focused on diagnosis.

While most studies attempted to involve people living with dementia as well as their family caregivers to identify research priorities, there remains limited evidence on the best approaches for engaging people with dementia and family caregivers from culturally and linguistically diverse backgrounds in research. This lack of representation echoed the findings of another recent systematic review^31^ which identified only 2 out of 18 studies involving people living with dementia from culturally diverse backgrounds. This is significant given that all the research included in this review was conducted in countries with high rates of incoming migration involving people from increasingly diverse ethnic, language, and cultural backgrounds.

There were, of course, calls for research on prevention, cure, and effective treatment, however, it is crucial to emphasise the importance of care-related topics for dementia as underscored by Armstrong et al^17^ and Kelly et al^24^ in their studies. Addressing the stigma and stereotype associated with dementia is imperative, as they hinder access to care and diminish the quality of life for people living with the condition and their caregivers. Research findings will contribute something to this but ideally, we need to work on changing public, private and workplace attitudes about dementia and encourage supporting and participating in dementia research. Health and aged care workforce education, training, and staffing have long been written about and criticised, including in the recent Australian Royal Commission on Aged Care Quality and Safety which was scathing.^32^ In our review, we observed a consistent call for more research and evidence from people living with dementia, caregivers, health professionals and the authors of these studies. The studies suggest that research is needed across all identified proprieties and funding should align with these priorities.^17,24,25^ Some studies also emphasise the need for more information about other potential research areas relevant to dementia.^19,23^ However, it is time to act on what is already known. We must move beyond conducting more research on the problem and instead direct our efforts towards implementing solutions. As highlighted by Shah et al,^27^ with established research priorities, we can now create a global plan for dementia aimed at reducing its global burden.

## Strengths and Limitations

Our study’s strength lies in the inclusion of findings predominantly from individuals living with dementia and family caregivers, complemented by insights from diverse stakeholders such as researchers, healthcare providers, clinicians, social care providers and community members. However, since most of the included studies involved additional stakeholders alongside people living with dementia and caregivers and did not stratify the results by stakeholder group, we were unable to examine if research priority topics identified by individuals with dementia and caregiver significantly differed from those identified by other stakeholder groups. Nevertheless, the studies noted that these groups supported and prioritised research topics that were also aligned with other stakeholder groups.^17,18^ Studies included in our review were conducted in Australia, Canada, the United Kingdom, and the United States. Priority areas for research identified in these countries may not be the same in other geographical regions due to different health systems and cultural approaches to health. As Logan et al^29^ reported, priorities varied by countries’ economic status, with higher priority for awareness and education in low- and middle-income countries, while high-income countries prioritised caregivers and support. Although an extensive search of the literature was conducted, we only included studies published in English. The inclusion of only English may have omitted important and relevant articles. Our study could also be limited by the search terms. While we took great care in creating search terms with support from a subject librarian and used individual and combinations of terms, it could be possible that our search terms may have missed relevant studies.

## Conclusion

This systematic review aimed to determine research priority settings from the perspective of people living with dementia and their caregivers. It was conducted and prepared in line with the Joanna Briggs Institute’s guidance for systematic reviews and evidence synthesis. While the research priorities and categories across the studies overlapped and encompassed a broad array of priorities, the desire for evidence-based research and solutions could be grouped into four areas: Increasing knowledge, education, and awareness; Determining cause; Sustainability of care; and Cure for dementia and related conditions. The high mortality and morbidity rates experienced by those living with dementia and the associated disease burden is only set to escalate, based upon the projected increase in the number of people diagnosed with dementia globally. It is critical, regardless of the difficulty experienced in providing a voice for those affected by this disease, that we act upon the research priorities thus far identified. Additionally, future research must include those from culturally and linguistically diverse communities focusing on their specific research priorities. The findings of this systematic review, provide some direction in terms of future research in this field, which is necessary not only to address access to care and quality of life for those people living with dementia but also to address the stigma associated with the diagnosis of dementia. This is particularly important considering recent findings demonstrating the challenges still faced in this care area.

## Supplemental Material


Supplemental Material - A Systematic Review of Dementia Research Priorities
Supplemental Material for A Systematic Review of Dementia Research Priorities by Manonita Ghosh, Pelden Chejor, Melanie Baker, and Davina Porock in Journal of Geriatric Psychiatry and Neurology.
